# Laryngotomy and Tracheotomy

**Published:** 1850-11

**Authors:** 


*Laryngotomy and Tracheotomy.*—The question whether the operation of laryngotomy or that of tracheotomy should be performed for the relief of asphyxia, resulting from obstruction of the larynx, whether that disease be acute, or consecutive to a chronic affection of the organ of voice, is of very considerable importance to the practical surgeon, and has been most satisfactorily answered in favor of laryngotomy, in a very interesting paper published by Mr. P. Hewett, in the *London Journal of Medicine*, for February, 1849. In this communication, Mr. Hewett states that, in all cases of acute inflammation of the larynx, in adults, and in those cases that occur in children, from swallowing boiling water or other irritating fluids, the obstruction is seated *above* the inferior vocal chords; the mucous membrane covering them, or that below them not being in the slightest degree thickened; and that consequently, as laryngotomy is a much simpler and safer operation than tracheotomy, it ought to be preferred in these cases.

John Erichson, Esq., states that he has been led to a similar conclusion in favor of laryngotomy by an inquiry into this subject, on which he entered some time since, and in the course of which he examined various recent specimens of inflamed and obstructed larynx, and those preparations of disease of this organ that are preserved in several of the pathological collections in London, without being able to meet with one in which an opening in the crico-thyroid membrane would not have relieved the asphyxia equally as well as one in the trachea. This examination determined him in future to have recourse to laryngotomy, rather than to tracheotomy, in acute obstructions of the glottis, whether primary or supervening in chronic ulceration or disease of the part. This determination was strengthened by the result of the post-mortem examination of two cases in which he had performed tracheotomy—the one, of old syphilitic disease of the larynx, followed by acute œdema; the other, of erysipelas of the glottis, in both of which the obstruction was found to be situated above the inferior vocal chords.

